# Enhancing spatio-temporal environmental analyses: A machine learning superpixel-based approach

**DOI:** 10.1016/j.heliyon.2024.e34711

**Published:** 2024-07-16

**Authors:** Enrique Estefania-Salazar, Eva Iglesias

**Affiliations:** CEIGRAM and Department of Agricultural Economics, Statistics and Business, Universidad Politécnica de Madrid, Madrid, 28040, Spain

**Keywords:** Environmental analysis, Big data, Dimension reduction, Machine learning, Superpixel, Vegetation indices

## Abstract

The progressive evolution of the spatial and temporal resolutions of Earth observation satellites has brought multiple benefits to scientific research. The increasing volume of data with higher frequencies and spatial resolutions offers precise and timely information, making it an invaluable tool for environmental analysis and enhanced decision-making. However, this presents a formidable challenge for large-scale environmental analyses and socioeconomic applications based on spatial time series, often compelling researchers to resort to lower-resolution imagery, which can introduce uncertainty and impact results. In response to this, our key contribution is a novel machine learning approach for dense geospatial time series rooted in superpixel segmentation, which serves as a preliminary step in mitigating the high dimensionality of data in large-scale applications. This approach, while effectively reducing dimensionality, preserves valuable information to the maximum extent, thereby substantially enhancing data accuracy and subsequent environmental analyses. This method was empirically applied within the context of a comprehensive case study encompassing the 2002–2022 period with 8-*d*-frequency-normalized difference vegetation index data at 250-m resolution in an area spanning 43,470 km^2^. The efficacy of this methodology was assessed through a comparative analysis, comparing our results with those derived from 1000-m-resolution satellite data and an existing superpixel algorithm for time series data. An evaluation of the time-series deviations revealed that using coarser-resolution pixels introduced an error that exceeded that of the proposed algorithm by 25 % and that the proposed methodology outperformed other algorithms by more than 9 %. Notably, this methodological innovation concurrently facilitates the aggregation of pixels sharing similar land-cover classifications, thus mitigating subpixel heterogeneity within the dataset. Further, the proposed methodology, which is used as a preprocessing step, improves the clustering of pixels according to their time series and can enhance large-scale environmental analyses across a wide range of applications.

## Highlights

• Spatio-temporal analyses pose a challenge in large-scale environmental studies.
• Low-resolution data impact environmental analyses' results.
• Our approach reduces data dimensionality with minimal information loss.
• Our superpixel algorithm provides larger benefits in heterogeneous areas.

## Graphical abstract

**Figure:**
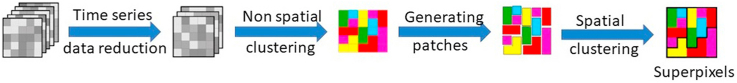

## 1 Introduction

Advances in remote sensing and satellite data have enabled large-scale dynamic environmental studies on various topics, including water pollution [1], water management [2], and land-use types [3]. Despite the vast potential of satellite images for studying extensive geographical areas, the full potential of remote sensing remains unrealized owing to the use of low-resolution (LR) pixel data when analyzing hyperspectral images or long time-series datasets. In such cases, researchers have often been compelled to employ LR pixels assuming subpixel homogeneity, primarily because of the high computational cost of using high-resolution (HR) pixels [4]. Subpixel heterogeneity within LR pixels is highly relevant because it introduces uncertainty and can undermine the accuracy and performance of models [5,6]. Moreover, the integration of remote sensing data into coarser-resolution images poses a challenge in terms of potential modeling results [7].

Substantial advancements in satellite technology, characterized by enhanced spatial and temporal resolutions and extended time series, pose an intricate challenge in the management of information. The significance of high-frequency (HF) data is underscored by its pivotal role in tracking dynamic processes. An equally critical issue is the demand for HR images, particularly in fields such as plant phenology [8]. Moreover, extended time series enable the exploration of long-term phenomena, offering unique insights into temporal dynamics [9]. However, reconciling these three essential attributes (high frequency (HF), HR, and long-term coverage) for large-scale analyses poses a formidable challenge due to difficulties in data processing. The integration of robust big data analysis tools is imperative to harness the full potential of HR and HF observations.

To address this challenge and develop computationally tractable algorithms by exploiting spatial correlation, superpixel segmentation has emerged as a widely adopted preprocessing technique [10]. Coined by Ren and Malik [11], a superpixel represents a grouping of pixels with shared attributes, such as color or other defining characteristics. Hence, superpixels facilitate the transition from pixel-to multipixel-level analyses, effectively reducing image complexity and allowing for more efficient environmental analyses. Originally, superpixels were designed for the computer vision domain and were restricted to RGB images; however, over time, superpixel segmentation has been applied to hyperspectral remote-sensing images [12].

Superpixel segmentation has shown widespread utility as a preprocessing step across the diverse domains of remote sensing and environmental quality management [13]. These applications include screening native species [14], monitoring water resource behavior changes [15], forest inventories and ecosystem conservation [16], lithological mapping [17], and cloud detection [18]. Furthermore, the increasing relevance of HR drone imagery presents new prospects for the application of superpixel algorithms [19].

The increasing interest in superpixels for environmental analyses, fueled by their multiple applications, has catalyzed the development of novel techniques and algorithms for their generation. Stutz et al. [20] conducted a comprehensive analysis of the 28 most commonly employed superpixel algorithms, which could be divided into five groups: watershed-, graph-, path-, cluster-, and wavelet-based. Different classifications can be used to distinguish between supervised and unsupervised learning methods. The distinguishing factor between these lies in the necessity for labels to train and validate the data of supervised methods, whereas unsupervised methods rely solely on the inherent information used for superpixel generation, leveraging these data to discern latent patterns [21].

As highlighted by Stutz et al. [20], contemporary superpixel algorithms, either supervised or unsupervised, rely primarily on a modest number of parameters that are often associated with image color attributes. However, a clear contrast arises when these algorithms encounter datasets characterized by a large number of parameters, such as those encountered in time-series analyses or hyperspectral images. Superpixel algorithms tailored to hyperspectral and time-series data remain notably underdeveloped compared with their color-based image segmentation counterparts, which have traditionally constituted their primary application. Existing superpixel algorithms geared towards time-series analyses tend to be either constrained to local applications because of their computationally intensive nature [22] or fall within the domain of supervised learning predominantly employed for classification purposes.

Many research articles have been published in the field of classification algorithms applied to time-series images, addressing various tasks, such as crop mapping, particularly rice cultivation [23], water surface detection [15], or land disturbance identification [24]. However, the quest for a superpixel algorithm that seamlessly integrates spatial time-series data with unsupervised learning techniques and exhibits practical feasibility at a large scale remains underdeveloped.

Gao et al. [25] proposed an unsupervised methodology grounded in the covariance between pixels, which is a promising approach that is cumbersome when applied to extensive regions. Zhou et al. [26] and Nowosad and Stepinski [27] addressed this issue and proposed an adaptation of Simple Linear Iterative Clustering (SLIC) [28] to time series data. The SLIC algorithm is based on a k-means clustering approach that uses RGB parameters as inputs and is one of the better-performing methodologies [20]. The main limitation of SLIC for time-series analyses is that it only admits three parameters. Nowosad and Stepinski [27] used a time series with 12 observations (monthly data for one year) in a 21,500-pixel grid and reduced the dimensions using principal component analysis (PCA). The first three principal components explained more than 99 % of the variance. However, when analyzing longer time series, the first three components rarely have sufficient explanatory power, generating suboptimal results. In addition, even if the first three components capture the most important pattern, they will not properly capture anomalies [29], which might be of interest for studying extreme environmental events, such as dry spells.

Consequently, when researchers aspire to conduct long time-series studies encompassing vast regions, they often need to resort to a lower resolution than the desired pixels or decrease the temporal frequency to address the challenges associated with data management [30]. This introduces subpixel noise into the dataset, which can significantly affect the performance of the subsequent analytical processes, particularly in heterogeneous areas [31].

In this paper, we present the Spatial Time Series-Based Superpixel (STiSeBS) algorithm, which is an innovative, computationally efficient, and time-saving unsupervised learning methodology designed to group similar pixels into superpixels based on satellite image time series. This work contributes to the literature addressing the challenge of large-scale spatio-temporal analyses by reducing the dimensionality of data with minimal information loss. Although we tested the algorithm using the Normalized-Difference Vegetation Index (NDVI), it was versatile and could be applied to several indices, or even hyperspectral images. In fact, the more heterogeneous the region or time series, the better the results derived from this methodology. Following the basic concept of the SLIC algorithm, our methodology integrates a k-means clustering approach and a regionalization algorithm with a prior dimensionality reduction step. The algorithm was tested in the Sahel Region of Burkina Faso, a large territory with an area of 43,470 km^2^. We leveraged a 20-year-time-series NDVI dataset (2002–2022), with an 8-d sampling frequency and a spatial resolution of 250 m. This case study showed that the proposed algorithm reduced the error by 25 % compared with low-resolution pixels and by 9 % compared with other algorithms.

The remainder of this paper is structured as follows. Section 2 describes our methodological approach and the assessment methods used to test the quality of the algorithm. Section 3 presents the results obtained in the case study, in which the methodology is tested, and evaluates its performance against LR pixels and the extended SLIC (e-SLIC) proposed by Nowosad and Stepinski [27], which is taken as a benchmark. Finally, Section 4 presents the conclusions of this study.

## 2 Materials and methods

**Fig. 1:**
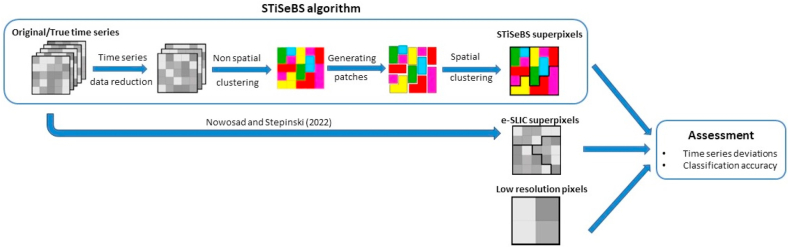
Process summary diagram.

The developed algorithm operates on the premise of aggregating pixels characterized by analogous time-series profiles using the Euclidean distance between the time series as the similarity measure. This method comprises four steps. First, we used PCA to reduce the time-series data to a smaller number of components. Subsequently, a non-spatially constrained k-means clustering algorithm was used to accelerate the process. Subsequently, contiguous pixels that belong to the same cluster and are contiguous are grouped into patches, and the patches are finally joined using a spatially constrained clustering algorithm. Each part of the algorithm is described in detail in the following sub-sections. Figure (1) shows a process summary diagram describing the STiSeBS algorithm and how the assessment is performed, comparing its performance with that of the LR pixels and e-SLIC superpixels.

### 2.1 Data acquisition and preprocessing

**Fig. 2:**
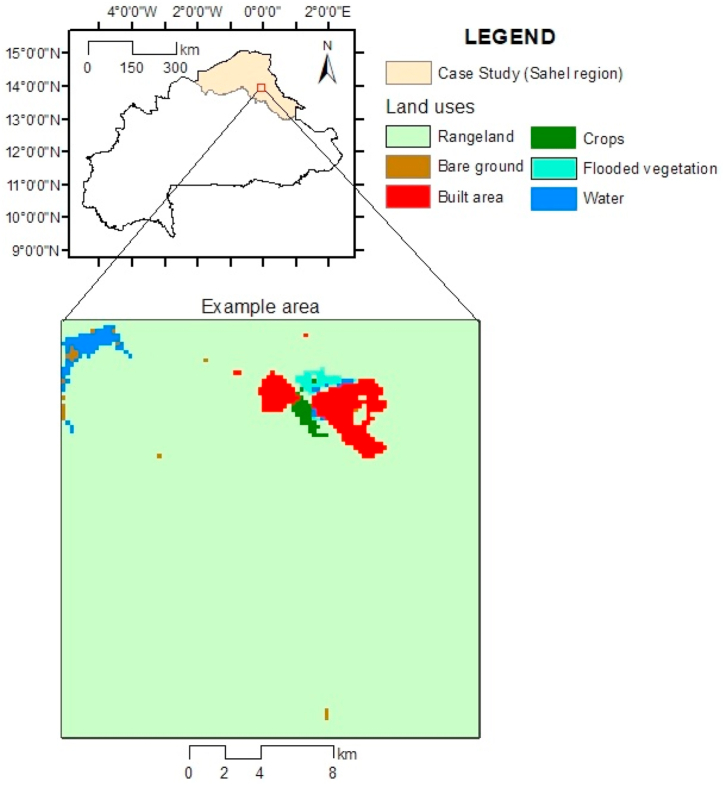
Location of the case study and example area in Burkina Faso.

NDVI time series data were acquired using the Application for Extracting and Exploring Analysis Reading Samples software (AppEEARS Team, 2023), which relies on observations from the Moderate-Resolution Imaging Spectroradiometer (MODIS) sensor aboard the Terra satellite. Our data retrieval encompassed red and near-infrared (NIR) bands with resolutions^1^ of 250 and 1000 m, respectively. We computed the NDVI values using Eq. (1). The time series, spanning March 31, 2002, to September 30, 2022, was characterized by an 8-d frequency, resulting in 944 periods (*T*). The method was evaluated within the Sahel region of Burkina Faso, which contained 695,532 HR pixels (*I*). This region is characterized by its predominance of rangelands, accounting for over 97 % of its land cover, and exhibits a notably uniform elevation, averaging approximately 300 m. The pastures within this area, which play a critical role in livestock production, are mainly arid or semi-arid (annual average NDVI<0.3).^2^ Figure (2) depicts the study's location within Burkina Faso and an example zone used for graphical clarification in the following subsections. To ensure data quality, we removed any NDVI values falling outside the [−1, 1] range, filled in any missing values using a three-window exponential weighted moving average, and performed Savitzky–Golay smoothing as previously described by Chen et al. [32].

(1) $${NDVI}_{i,t}=\frac{{NIR}_{i,t}-{Red}_{i,t}}{{NIR}_{i,t}+{Red}_{i,t}}$$

where subindex *i* = 1, …,I refers to each pixel and *t* = 1, …, T refers to the time periods.

### 2.2 Time-series data reduction

To enhance the computational efficiency, especially to accommodate HF frequency and HR images and more extensive time-series datasets in the future, it is imperative to reduce the dimensionality of the dataset, represented by the number of time periods. Consequently, a PCA was performed, retaining the first components that collectively explained 99 % of the variance in the dataset. For this case study, the first 200 components, which corresponded to the linear combinations that optimized the explained variance, were selected using the Lagrangian method, as indicated in Eqs (2), (3), (4). Consequently, the database dimensions were reduced from 944 time periods to 200 components. The number of components can be flexibly adjusted to strike a balance between computational efficiency and analytical precision.

(2) $$\max_{w}\;w^{T\;}Cw$$

(3) $$s.t.\;w^{T}w=1$$

(4) $$L\left(w,\lambda \right)=w^{T\;}Cw-\lambda \left(w^{T}w-1\right)$$

where *w* represents the projection axes matrix IxT (eigenvectors of *C*), *C=XX*^*T*^ is the covariance matrix IxI*,* and *λ* is the vector Tx1 of the corresponding eigenvalues of *w*.

### 2.3 Non-spatially constrained clustering (k-means)

After reducing the time-series data using PCA, we performed spatial dimension reduction. Similar to the SLIC algorithm, the proposed methodology relies on the k-means clustering approach. The k-means algorithm, originally introduced by Lloyd [33], randomly initializes a quantity of C centroids equivalent to that of the specified number of clusters. Subsequently, each data point is assigned to its nearest centroid, which minimizes the objective function, as shown in Eq. (5). Successive iterations and new centroids were computed until the centroids no longer underwent significant changes. Although more advanced clustering techniques, such as Density Based Spatial Clustering of Applications with Noise, Gaussian mixture models, and spectral clustering, may be considered as alternatives to k-means clustering, their application would substantially increase the computational demand, potentially rendering them unfeasible for extensive geographic regions when using conventional computing platforms.

(5) $$\min\;\sum_{q\left(x_{i}\right)=r}{\left\|C_{r}-x_{i}\right\|}^{2}$$

where *q*(*x*_i_) is a function that returns the centroid to which pixel *i* belongs, *x*_i_ is the vector of components of pixel *i*, and *C*_r_ is the vector of components of the centroid to which pixel *i* belongs.

**Fig. 3:**
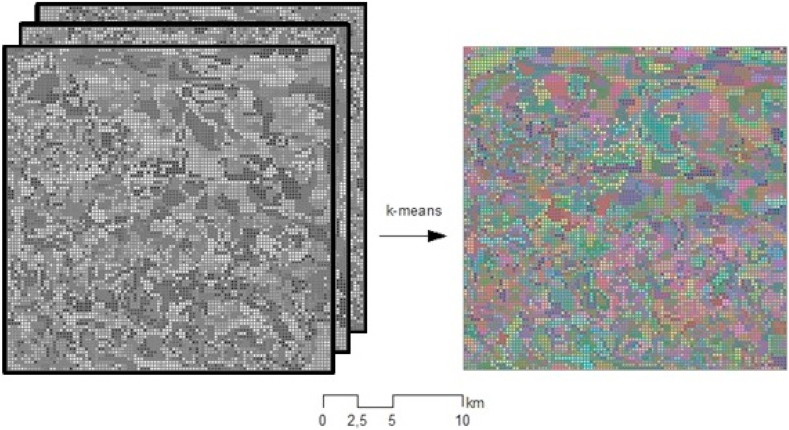
k-means clustering in the example area.

We performed k-means clustering by setting the number of clusters^3^ to 43,470 (Fig. 3).

### 2.4 Generating patches

**Fig. 4:**
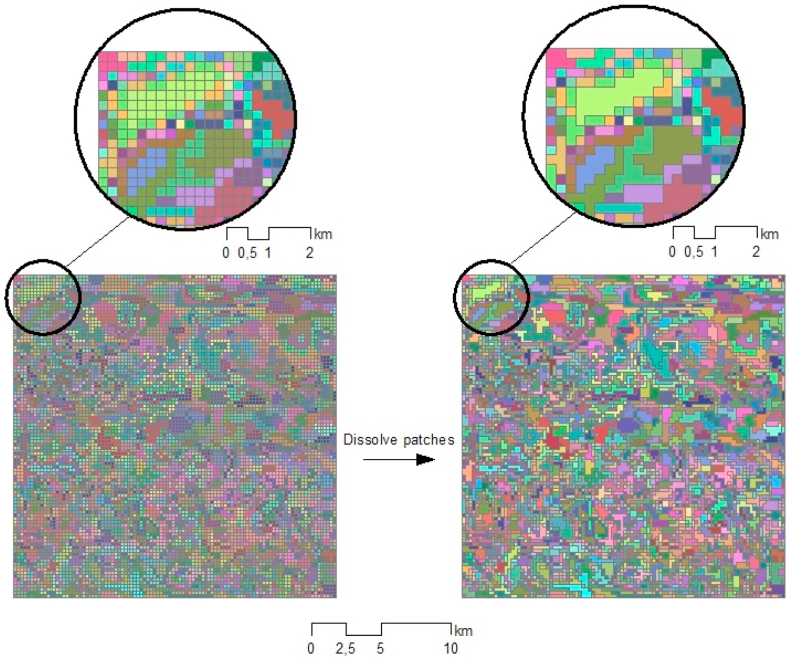
Patch dissolution in the example area.

After k-means clustering, contiguous pixels belonging to the same cluster were grouped to form 294,743 patches (*J*) (Fig. 4). Subsequently, we assigned the average principal component value derived from the constituent pixels to each patch (*j*), as shown in Eq. (6).

(6) $$x_{j}=\frac{1}{H}\sum_{i'=1}^{H}x_{i'}\;\forall \;i'\in j$$

where *H* is the number of pixels on a patch and *x*_*j*_ is the vector of components of patch *j*.

### 2.5 Spatially constrained clustering

Next, we grouped the patches into superpixels. Two main spatially constrained clustering algorithms that minimized the Euclidean distance between components emerged as viable alternatives, with their main differences residing in the inputs required by each one. The Max-p clustering algorithm [34] does not require the determination of the number of clusters in advance; therefore, the number of generated superpixels cannot be controlled. Thus, we selected a second algorithm, the Spatial “K”lustering Analysis by Tree Edge Removal (SKATER) clustering [35]. This method enables the selection of the desired number of superpixels and demonstrates remarkable efficiency, particularly when dealing with large datasets [36].

The SKATER algorithm first constructs a connectivity graph among objects (patches), captures their adjacency relationships, and assigns a similarity value to each connection as designated by the Euclidean distance between the principal components. To streamline computational efficiency, this connectivity graph was transformed into a minimum-spanning tree using Prim's algorithm [37]. The minimum-spanning tree is subjected to iterative pruning to yield two distinct trees. The pruning operation targets the edges that minimize the sum of the intracluster square deviations, as defined in Eq. (7).

(7) $${SSD}_{k}=\frac{1}{n_{k}}\sum_{j'=1}^{n_{k}}{\left(x_{j'}-x_{k}\right)}^{2}\;\forall \;j'\in k$$

where *SSD*_k_ represents the sum of the intracluster square deviations for the *k* tree (cluster or superpixel), *n*_k_ is the number of elements in a tree (patches), *x*_j′_ is the vector of components of patch *j′*, and *x*_k_ is the vector of components of superpixel *k.*

**Fig. 5:**
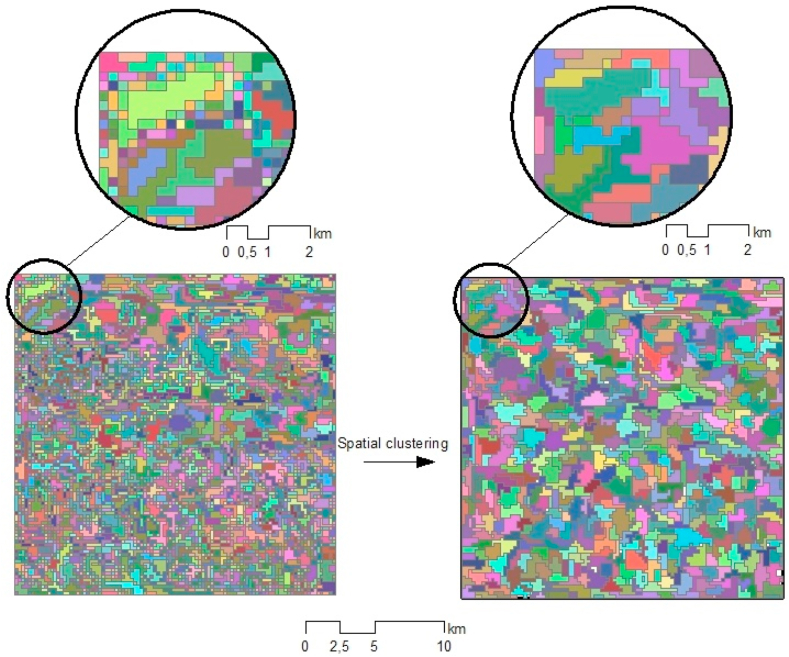
Spatial clustering in the example area.

As our methodology aims to be assessed by comparing its results with 1000-m-resolution pixels (and with superpixels derived from the e-SLIC algorithm), an equal number of superpixels to that of the LR pixels in the region was created, resulting in *k* = 43,470 (Fig. 5). After performing SKATER clustering, the NDVI time series for each superpixel *k* corresponded to the average value of all the pixels that formed it, as shown in Eq. (8).

(8) $${NDVI}_{k}=\frac{1}{H}\sum_{i'=1}^{H}NDVI_{i'}\;\forall \;i'\in s$$

where.

*H* is the number of pixels in the superpixel, and *NDVI*_*i′*_ and *NDVI*_*k*_ are the vectors of the NDVI values of pixel *i′* and superpixel *k*, respectively.

The entire process was executed on a conventional computer with 16 GB of RAM and an “AMD Ryzen 7 5800H″ processor, and took 30 h to complete.

### 2.6 Assessment methods

**Fig. 6:**
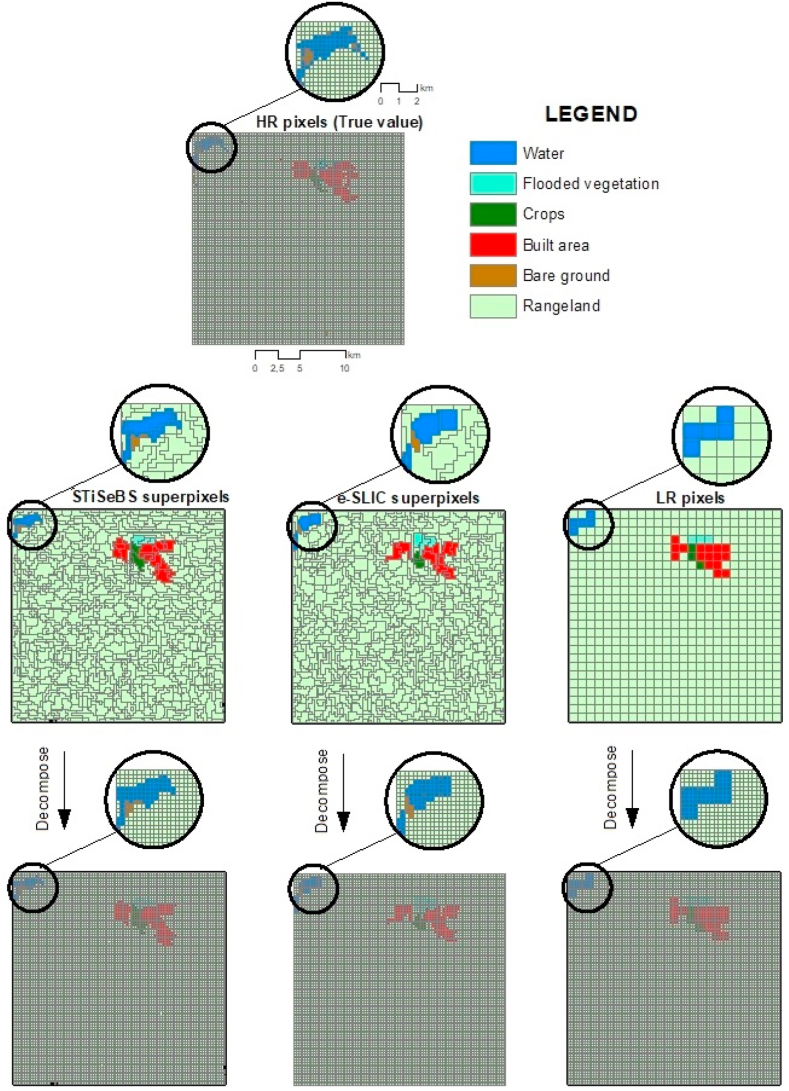
Assessment flowchart.

We assessed the performance of the STiSeBS algorithm based on the spatial NDVI time-series data of the case study area. We assumed that the HR pixels (250-m grid) represented the ground truth and compared the performance of the STiSeBS algorithm against the LR pixels and e-SLIC algorithm-based superpixels (we generated 43,470 e-SLIC-based superpixels following Nowosad and Stepinski [27]). We also evaluated its ability to group pixels based on land use. To achieve this, the STiSeBS and e-SLIC superpixels and LR pixels were disassembled into their constituent hypothetical HR pixels and subsequently assigned the values of their corresponding superpixels or LR pixels, as shown in Fig. 6.

#### 2.6.1 Time-series deviations

We assessed the accuracy of the STiSeBS algorithm by comparing the Euclidean distances between the NDVI time series of the true HR pixels and that of the hypothetical HR pixels derived from the STiSeBS, e-SLIC superpixels, and LR pixels, as previously described by Fei et al. [38] (Eq. (9)).

(9) $$Euclidean\;error=\sum_{i=1}^{I}\sum_{t=1}^{T}\sqrt{\hat{{NDVI}_{it}-}{NDVI}_{it}}$$

where.

$\hat{{NDVI}_{it}}$ represents the estimated NDVI value for pixel *i* in period *t* based on the corresponding superpixel (LR pixel) NDVI value, and ${NDVI}_{it}$ is the true NDVI value of pixel *i* in period *t*.

Jasiewicz et al. [39] proposed two validation assessment metrics: inhomogeneity (δ) and isolation (γ). δ describes the dissimilarity among pixels that belong to the same superpixel or LR pixel and is defined as the average distance between all the subunits (pixels) that constitute a larger unit (superpixel or LR pixel). The γ metric is the average distance between a given superpixel (LR pixel) and all of its neighbors. The smaller and larger that δ and γ are, respectively, the better the superpixels (LR pixels) will be. We used the Euclidean distance between time series as a distance metric.

#### 2.6.2 Classification accuracy

Subpixel heterogeneity is one of the most important challenges in environmental modeling [5]. Although the algorithm was designed to group pixels with similar time series, it also indirectly combined HR pixels of the same class. To highlight this noteworthy effect, we leveraged the 2021 land-cover map derived from Sentinel-2 satellite data [40]. Within this context, we assigned land-use categories to each superpixel and LR pixel based on the most represented category among their constituent pixels. Our primary objective was to evaluate the ability of the STiSeBS algorithm to cluster pixels sharing identical land-use attributes, particularly in contrast to e-SLIC and LR pixels, which tended to exhibit higher heterogeneities. We generated hypothetical HR pixels and assigned them the value of the superpixel or LR pixel to which they belonged. The true and predicted land covers were compared using accuracy measures delineated by Congalton [41] and Zhang et al. [42] (see Eqs (10), (11))) and confusion matrix-based metrics, as in Fei et al. [38] and Yang et al. [15] (Eqs (12), (13), (14), (15)).

(10) $$Overall\;accuracy\;\left(OA\right)=\frac{TP+TN}{TP+TN+FP+FN}$$

(11) $$Kappa\;coefficient=\frac{I^{2}\cdot OA-\sum_{l=1}^{L}a_{l}\cdot b_{l}}{I^{2}-\sum_{l=1}^{L}a_{l}\cdot b_{l}}$$

(12) $$Precision=\frac{True\;positive}{True\;positive+False\;positive}$$

(13) $$Sensitivity=\frac{True\;positive}{True\;positive+False\;negative}$$

(14) $$F_{1}=2\;\frac{Precision\cdot Sensitivity\;}{Precision+Sensitivity}$$

(15) $$MCC=\frac{TP\cdot TN-FP\cdot FN\;}{\sqrt{\left(TP+FP\right)\left(TP+FN\right)\left(TN+FP\right)\left(TN+FN\right)}}$$

where.

TP is a true positive, TN is a true negative, FP is a false positive, and FN is a false negative; a_l_ is the actual number of pixels for each land use type; b_l_ is the predicted number of pixels for each land use type; and MCC is the Matthews correlation coefficient.

## 3 Results and discussion

### 3.1 Time-series deviations

The cumulative Euclidean error incurred by the StiSeBS algorithm across the entire region was 8,464,205, whereas the e-SLIC superpixels and LR pixels yielded errors of 9,272,029 and 10,601,484, respectively. This discrepancy underscores the substantial advantages of employing the proposed methodology over the utilization of LR pixels, leading to a 25.25 % reduction in error. Moreover, the StiSeBS algorithm enhanced the performance of the benchmark e-SLIC by 9.54 %. Given the homogeneity of the area, the three principal components of the time series accounted for 80 % of the variance and represented a notable level of explanatory power. Additionally, the interannual cyclical pattern observed in the NDVI introduced redundancy to the time series, thereby decreasing the number of principal components required to explain a substantial portion of the variance in the dataset (the first three components explained the seasonal pattern, but may not have properly captured intraseasonal specificities). Therefore, in this case study, our methodology did not yield superior results compared with the benchmark. The difference in error was due to the characteristics of the time series unaccounted for 80 % of the variance (the first three components). These differences were mainly peculiarities or anomalies in the time series, which could be especially important if the objective of the subsequent environmental analyses was related to extreme or infrequent events such as dry spells. Therefore, STiSeBS is preferable in these cases. In addition, it is anticipated that, in more heterogeneous datasets, either because of the idiosyncrasies of the index (or hyperspectral images) or the diversity of the study area, our methodology could produce much better results than the benchmark.

**Fig. 7:**
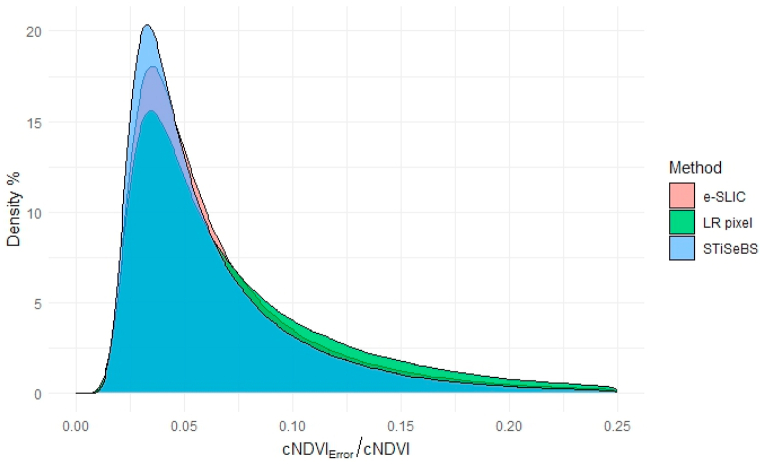
Per-pixel NDVI error density plot for each method. Note: cNDVI_Error_ is the cumulative error established along the whole time series and cNDVI is the true cumulative NDVI.

Fig. 7 shows a density plot of the cumulative committed error in the NDVI time-series prediction for each pixel, depending on the method. We performed the Kolmogorov–Smirnov test and found statistically significant differences among the three distributions (p < 0.01). The STiSeBS algorithm outperformed the benchmark method and LR pixels, which are the most frequently used inputs for large-scale environmental analyses. It was less skewed toward the positive side and had a higher proportion of low-error pixels.

Table 1 lists the inhomogeneity and isolation metrics for each methodology. STiSeBS-based superpixels were the most homogeneous, meaning that the pixels within them had similar time-series patterns. LR pixels performed worse than the benchmark algorithms. Although further research is needed, it is expected that in a more heterogeneous area, the first three components will explain less of the variance, and the e-SLIC-based superpixels are expected to be more dissimilar to the STiSeBS-based superpixels in terms of homogeneity. Remarkably, the superpixels delineated using the e-SLIC algorithm were more different than those delineated using STiSeBS (lower isolation). However, as highlighted by Nowosad and Stepinski [27] superpixels do not necessarily differ. From this result, we can conclude that we can reduce the number of STiSeBS-based superpixels without notably increasing the error (neighboring superpixels are quite similar; therefore, we can group them). Although not the objective of this study, these metrics can be used to determine the optimal number of superpixels in a given region.

**Table 1:** Time series performance metrics.

Method	Inhomogeneity	Isolation
STiSeBS	5.629	1.034
e-SLIC	6.427	1.178
LR pixels	7.021	0.965

### 3.2 Classification accuracy

The overall accuracy achieved using the STiSeBS approach was 97.79 %, whereas the LR pixels and the benchmark methodology yielded accuracies of 97.73 % and 97.76 %, respectively. These elevated accuracy values were influenced by the predominance of the “rangelands” land-cover type, which accounted for 97 % of the total pixels. To provide a more rigorous assessment, we estimated additional metrics. The kappa coefficient primarily emerged as a valuable indicator, with values of 0.491, 0.482, and 0.465 for the STiSeBS, e-SLIC, and LR pixels, respectively. This sophisticated metric underscores the capacity of the STiSeBS algorithm to effectively group pixels belonging to the same land-cover category. To further scrutinize the algorithm's performance in discerning individual land cover types, we constructed confusion matrices for the STiSeBS, e-SLIC, and LR pixels across all land-cover categories (Table 2, Table 3, Table 4).

**Table 2:** Confusion matrix for the STiSeBS based HR pixels.

Land use type	TP	FP	FN	TN	Precision	Sensitivity	F score	MCC
Water	268	91	343	694,830	0.747	0.439	0.553	0.572
Trees	2456	1085	5515	686,476	0.694	0.308	0.427	0.458
Flooded vegetation	155	41	78	695,258	0.791	0.665	0.723	0.725
Crops	572	256	669	694,035	0.691	0.461	0.553	0.564
Built area	679	184	342	694,327	0.787	0.665	0.721	0.723
Bare ground	3361	1318	5570	685,283	0.718	0.376	0.494	0.516
Rangeland	672,738	12,328	2786	7680	0.982	0.996	0.989	0.521

**Table 3:** Confusion matrix for the e-SLIC based HR pixels.

Land use type	TP	FP	FN	TN	Precision	Sensitivity	F score	MCC
Water	236	114	375	694,807	0.674	0.386	0.491	0.510
Trees	2589	1174	5389	686,380	0.688	0.325	0.441	0.469
Flooded vegetation	147	101	86	695,198	0.593	0.631	0.611	0.611
Crops	581	266	663	694,022	0.686	0.467	0.556	0.565
Built area	643	207	378	694,304	0.756	0.630	0.687	0.690
Bare ground	2956	1016	5979	685,581	0.744	0.331	0.458	0.492
Rangeland	672,828	12,674	2682	7348	0.982	0.996	0.989	0.509

**Table 4:** Confusion matrix for the LR based HR pixels.

Land use type	TP	FP	FN	TN	Precision	Sensitivity	F score	MCC
Water	175	113	436	694,808	0.608	0.286	0.389	0.417
Trees	2050	1085	5921	686,476	0.654	0.257	0.369	0.406
Flooded vegetation	105	55	128	695,244	0.656	0.451	0.534	0.544
Crops	616	259	625	694,032	0.704	0.496	0.582	0.591
Built area	613	155	408	694,356	0.798	0.600	0.685	0.692
Bare ground	3408	1234	5523	685,367	0.734	0.382	0.502	0.525
Rangeland	672,784	12,880	2740	7128	0.981	0.996	0.989	0.498

Generally, in terms of the F-score and MCC, the STiSeBS algorithm yielded better results than the other two methodologies for almost all categories. The most substantial improvements were observed for “water” and “flooded vegetation” land-cover types, which were the most infrequent (0.08 % and 0.03 %, respectively). The first three principal components failed to sufficiently explain the temporal dynamics of underrepresented observations, resulting in a discrepancy between their representation in three-dimensional space and the original multidimensional time series. That is, the first principal component ignored the idiosyncrasies of infrequent land cover. Therefore, the STiSeBS algorithm performed better than the e-SLIC algorithm in the classification of poorly represented land cover types. The same argument can be extrapolated to outliers in terms of the time series. This implies that STiSeBS can better detect outlier zones and group those pixels into homogeneous superpixels.

Comparing the two superpixel-based methodologies, the STiSeBS algorithm only performed slightly worse than the benchmark when grouping “trees” (MCC of 0.458 and 0.469 respectively). This land category accounted for more than 1 % of the pixels, and its time series had a very different pattern to that of rangelands (the most frequent category). In this case, the first three principal component approaches accurately reflected the characteristics of forests.

Both superpixel-based methodologies generated worse results than arbitrary LR pixels when attempting to group “crop” pixels. This may be because of two reasons. First, there may be crops with different phenological patterns. Therefore, when joining pixels according to their time series, they are not grouped correctly because they do not exhibit similar time-series patterns. That is, within the land category “crop”, there are very different pixels. Second, the results may be affected by land-use changes because the land-cover map was based on the 2021 Copernicus land cover, whereas the clustering was based on 20 years of NDVI data. The expansion of the agricultural frontier in the study area [43] has resulted in more and more pixels being considered “crops” when years ago they would not have been. Regarding the confusion matrices (Table 2, Table 3, Table 4), the poor performance of the superpixel-based methodologies arose from the higher number of false negatives, which could have indicated that these algorithms were detecting “crops” of 2021 as non-crops during the time series, which is consistent with the expansion of the agricultural frontier. However, future research should explore the fact that even random grouping, such as LR pixels, yields superior outcomes compared with the two methodologies rooted in time-series analysis.

## 4 Conclusions

Large amounts of spatiotemporal remote sensing data enable countless applications, but pose a challenge in terms of data manageability, especially when dealing with large areas, which is a common feature in many environmental studies. In this study, we proposed a methodology for grouping pixels with similar time-series patterns to reduce the spatial dimensions of large spatiotemporal databases. The developed STiSeBS algorithm groups pixels according to the time-series similarities of higher-resolution images and outperforms other superpixel algorithms for time-series and lower-resolution images, which are frequently used to avoid data manageability issues in large-scale environmental analyses. Using 20-year NDVI data from a 43,470-km^2^ area as a case study, our methodology improved the total cumulative deviation in the time series by 9 % with respect to the benchmark methodology (extended SLIC). Moreover, the STiSeBS algorithm demonstrated better performance in grouping pixels with the same land cover and was especially valuable for detecting less frequent land use types. Finally, because it did not rely on a limited number of parameters (principal components in the case of extended SLIC), it may be particularly helpful for large heterogeneous areas and for indices with complex dynamic patterns, hyperspectral images, or for grouping pixels that suffer from anomalies jointly (e.g., extreme droughts).

## Data availability statement

Data included in the article are referenced in it.

## CRediT authorship contribution statement

**Enrique Estefania-Salazar:** Writing – original draft, Visualization, Software, Methodology, Investigation, Funding acquisition, Formal analysis, Data curation, Conceptualization. **Eva Iglesias:** Writing – review & editing, Validation, Supervision, Resources, Project administration, Methodology, Investigation, Formal analysis, Conceptualization.

## Declaration of competing interest

The authors declare that they have no known competing financial interests or personal relationships that could have appeared to influence the work reported in this paper.

## Footnotes

1 We refer to 250 m as HR, although it cannot be considered as HR, to distinguish it from the coarser-resolution 1000-m pixels. The complete analysis could have been conducted with two different resolutions, but we wanted to use data derived from the same satellite to avoid bias.

2 We could have chosen any large-scale area for the comparison, but we chose this area due to its high degree of homogeneity because we wanted to investigate whether the STiSeBS, even if not fully leveraged compared with the e-SLIC, could still improve its performance.

3 As we aimed to compare the performance of the algorithm with a 1000-m-resolution grid, which contained 16 HR pixels, we grouped 695,532 pixels into superpixels containing 16 pixels on average.
